# Concurrent Abdominal and Mediastinal Hydatid Cystic Disease: A Rare Case

**DOI:** 10.7759/cureus.55695

**Published:** 2024-03-06

**Authors:** Randeep Singh, Sidharth Garg, Pavneet Sidhu, Savijot Singh, Sameer Singh Faujdar, Raminderjit Singh

**Affiliations:** 1 General Surgery, Maharishi Markandeshwar Medical College and Hospital, Solan, IND; 2 Cardiovascular and Thoracic Surgery, Government Medical College & Hospital, Chandigarh, Chandigarh, IND; 3 Biochemistry, Adesh Medical College and Hospital, Shahbad, IND; 4 General Surgery, Adesh Medical College and Hospital, Shahbad, IND; 5 Microbiology, Maharishi Markandeshwar Medical College and Hospital, Solan, IND; 6 General Medicine, New Cross Hospital, Wolverhampton, GBR

**Keywords:** mediastinal hydatid, lung hydatid cyst, helminths, echinococcus, hydatid disease

## Abstract

Hydatid disease in humans is caused by accidental ingestion of *Echinococcus *in its larval form. It mostly affects the liver and lungs, but rarely the mediastinum and other areas as well. The diagnosis is mostly confirmed intraoperatively in cases of mediastinal disease. The mainstay of treatment in such cases is surgery. This is a case report of a rare finding of hydatid disease in mediastinum along with the abdomen and its surgical management.

## Introduction

Human hydatid disease is a zoonotic infection caused by accidental ingestion of the larval form of *Echinococcus granulosus* and is a common helminthic disease worldwide. After entering the blood circulation, they colonize various organ systems of the body. The most commonly affected areas are the liver and lungs, but the extrapulmonary location of hydatid disease in the thorax is very rare. Intrathoracic extrapulmonary locations involve the mediastinum, pleura, pericardium, and chest wall. Mediastinal hydatidosis is rare and has an incidence of 0.1-0.5%. Mediastinal hydatid cysts are difficult to distinguish from other mediastinal cystic lesions and diagnosis is only made during surgery. Concurrent abdominal and mediastinal hydatid cysts are a very rare entity. Various serological tests have been proposed to establish the diagnosis of the disease and their judicious use may confirm the diagnosis in 80-94% of hepatic hydatidosis and 65% of pulmonary hydatidosis. Ultrasonography, computed tomography (CT), and magnetic resonance imaging (MRI) help establish the diagnosis. Signs and symptoms of the cysts depend on their site and size. Large cysts can compress the vital organs and are responsible for pressure symptoms. Patients might present with retrosternal pain, cough, and shortness of breath in case of lung hydatidosis and abdominal pain, palpable mass, and abdominal distension in case of hepatic hydatidosis. Radical surgical excision is the gold standard of therapy and involves the removal of germinative membrane and pericyst. The role of albendazole is controversial [1]. We report a rare case of concurrent abdominal and mediastinal hydatid in a 60-year-old male.

## Case presentation

**Figure 1:**
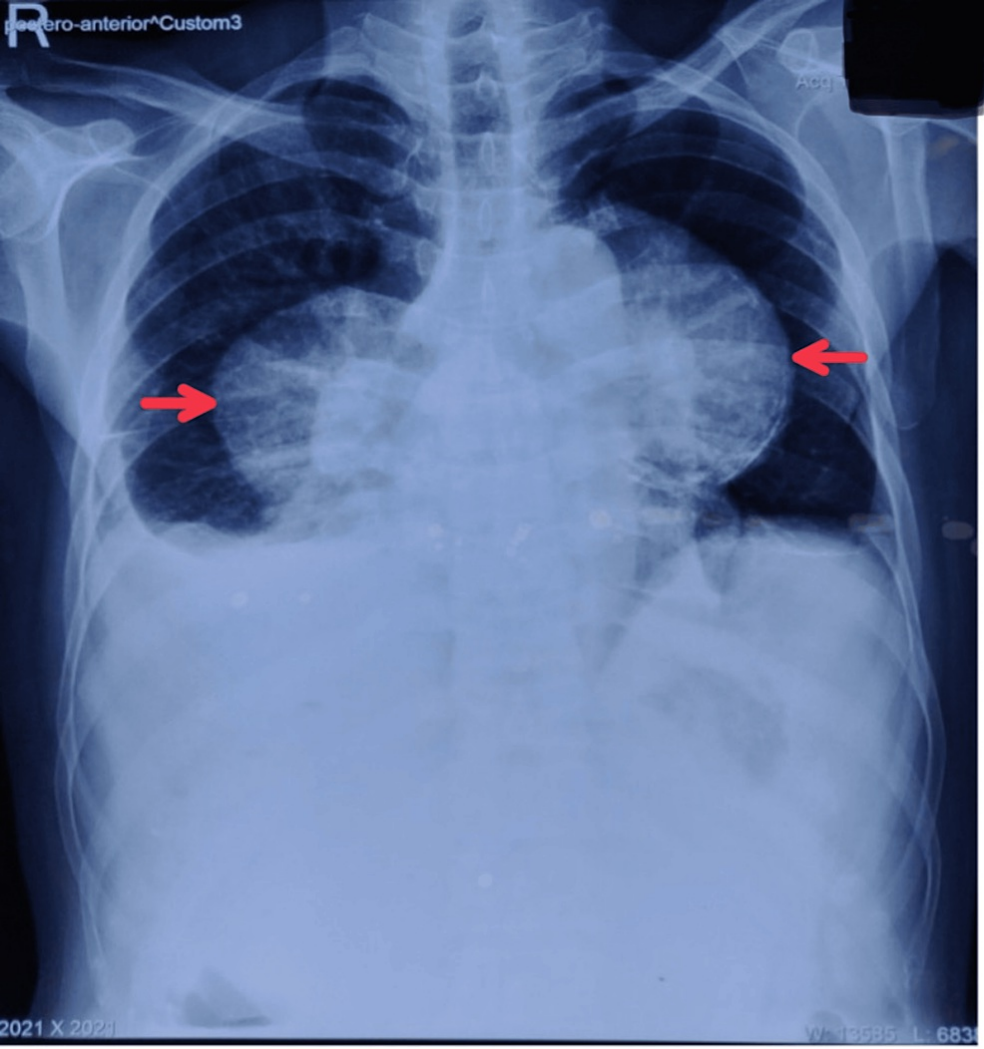
Chest X-ray suggestive of large masses in the anterior mediastinum.

A 59-year-old male presented to the outpatient department with a one-month history of dull aching, non-radiating, non-shifting abdominal pain localized to the right hypochondrium. It was associated with abdominal distension and swelling in bilateral lower limbs. The patient also complained of chronic cough and shortness of breath, which exaggerated while doing strenuous work and climbing stairs. The patient was a laborer by profession and a chronic smoker for 30 years. The patient had no comorbidities and had no surgery in the past. On clinical examination, there was a vague palpable mass in the right hypochondrium with abdominal distension and no guarding or rigidity. Blood workup was suggestive of raised alkaline phosphatase (147 IU/L), normal aspartate transaminase (serum glutamic-oxaloacetic transaminase)/alanine transaminase (serum glutamic pyruvic transaminase) levels (49/64 IU/L), and normal bilirubin levels (0.6 mg/dL). Chest X-ray was suggestive of large masses in the anterior mediastinum (Figure 1).

**Figure 2:**
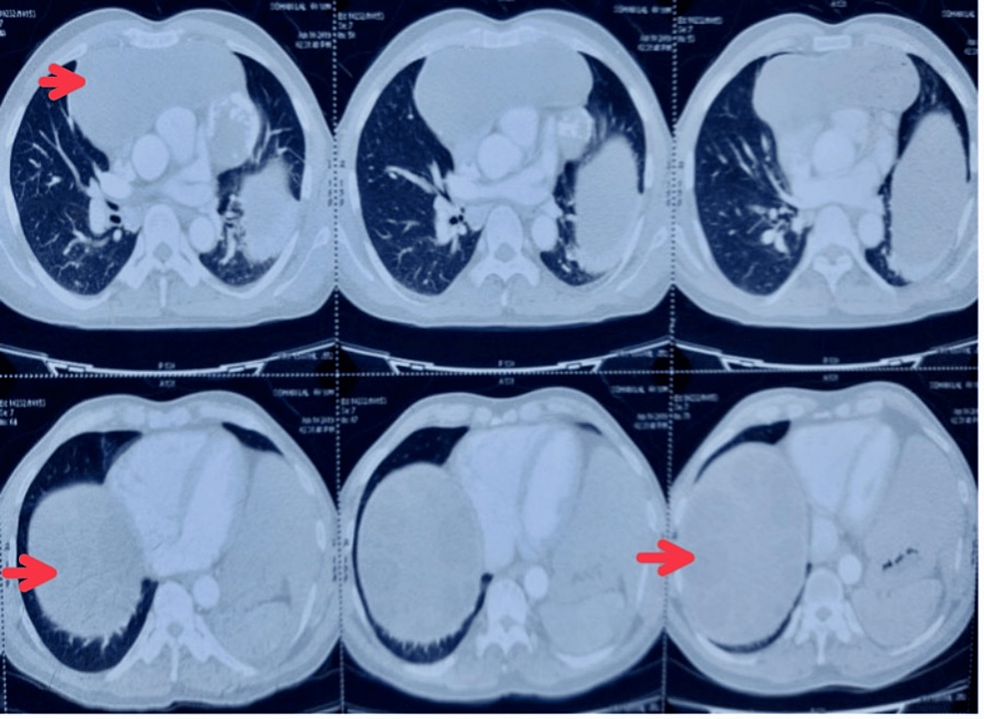
Contrast-enhanced computed tomography suggestive of hydatid cyst in the anterior mediastinum and the right lobe of the liver.

Ultrasonography was suggestive of hepatomegaly with a large well-defined heterogeneous cystic lesion with multiple peripheral daughter cysts suggestive of hydatid cyst with splenomegaly. Contrast-enhanced CT was suggestive of a large hypodense cystic lesion with peripheral calcification with internal daughter cysts (190 mm × 88 mm × 132 mm) suggestive of hydatid cyst in the anterior mediastinum with a similar lesion (155 mm × 62 mm) in the right lobe of the liver (Figure 2).

**Figure 3:**
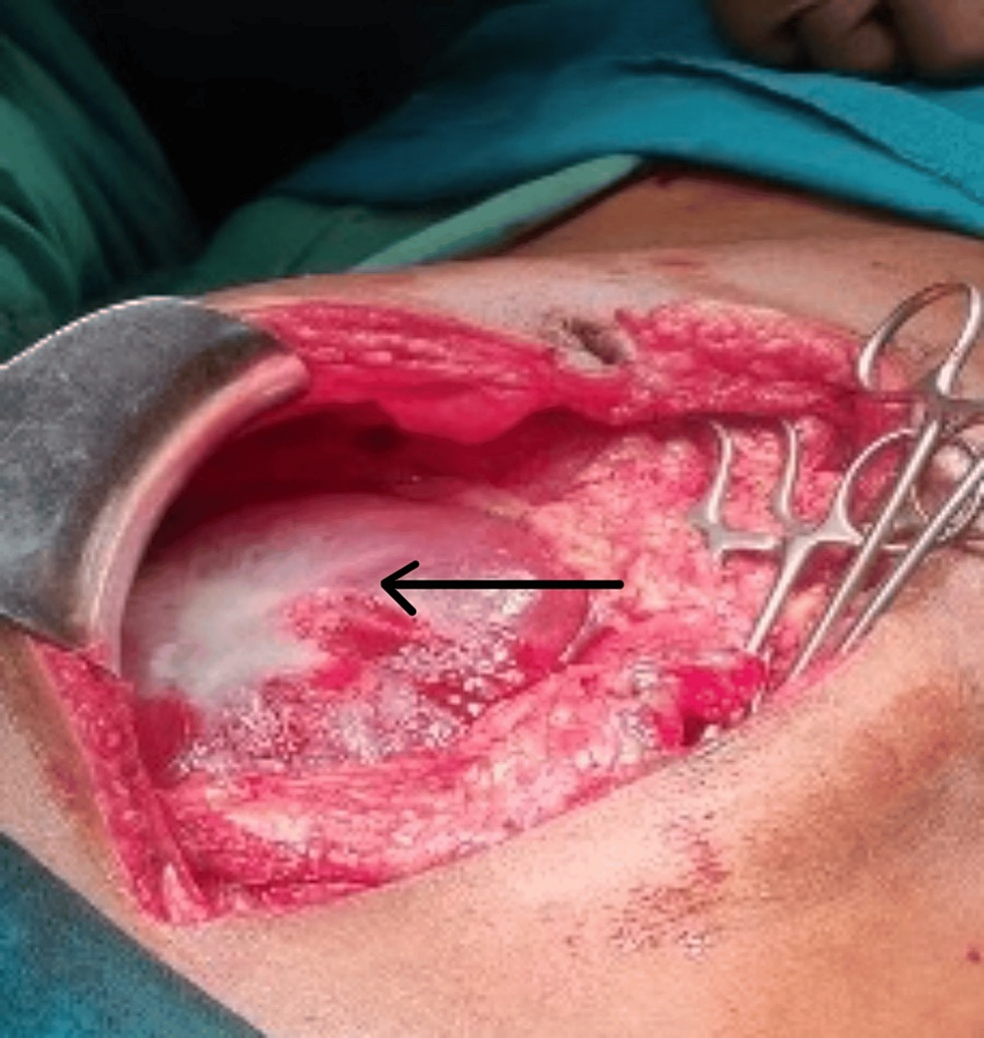
Intraoperative view of hydatid cyst in the liver.

**Figure 4:**
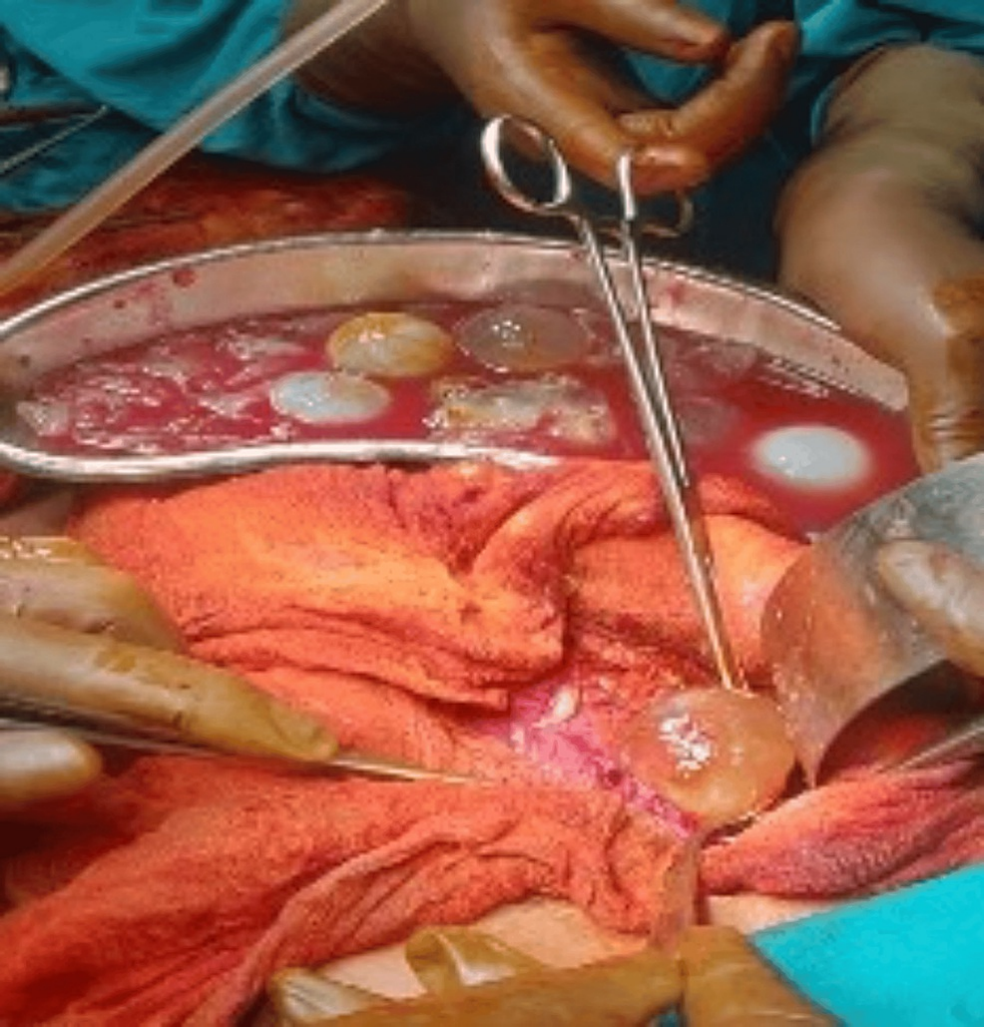
Intraoperative view of daughter cysts.

The patient was then planned for a two-staged surgery. In the first stage, the patient underwent exploratory laparotomy with marsupialization and daughter cyst extraction. Intraoperatively, there was a 10 cm × 15 cm cavity present in the right lobe of the liver, and the left lobe was found to be hypertrophied (Figures 3, 4).

After the first surgery, aspirated cyst fluid was sent to the microbiological laboratory for the demonstration of hydatid elements. The centrifuged hydatid fluid was examined by the wet mount method and modified Ziehl-Neelsen staining. Both were positive for hooklets and protoscolices of *E. granulosus*.

Postoperatively, the patient was kept in intensive care unit for hemodynamic monitoring and in view of poor respiratory efforts. The patient was discharged after nine days of hospital stay without any complications and was followed up in the outpatient department.

**Figure 5:**
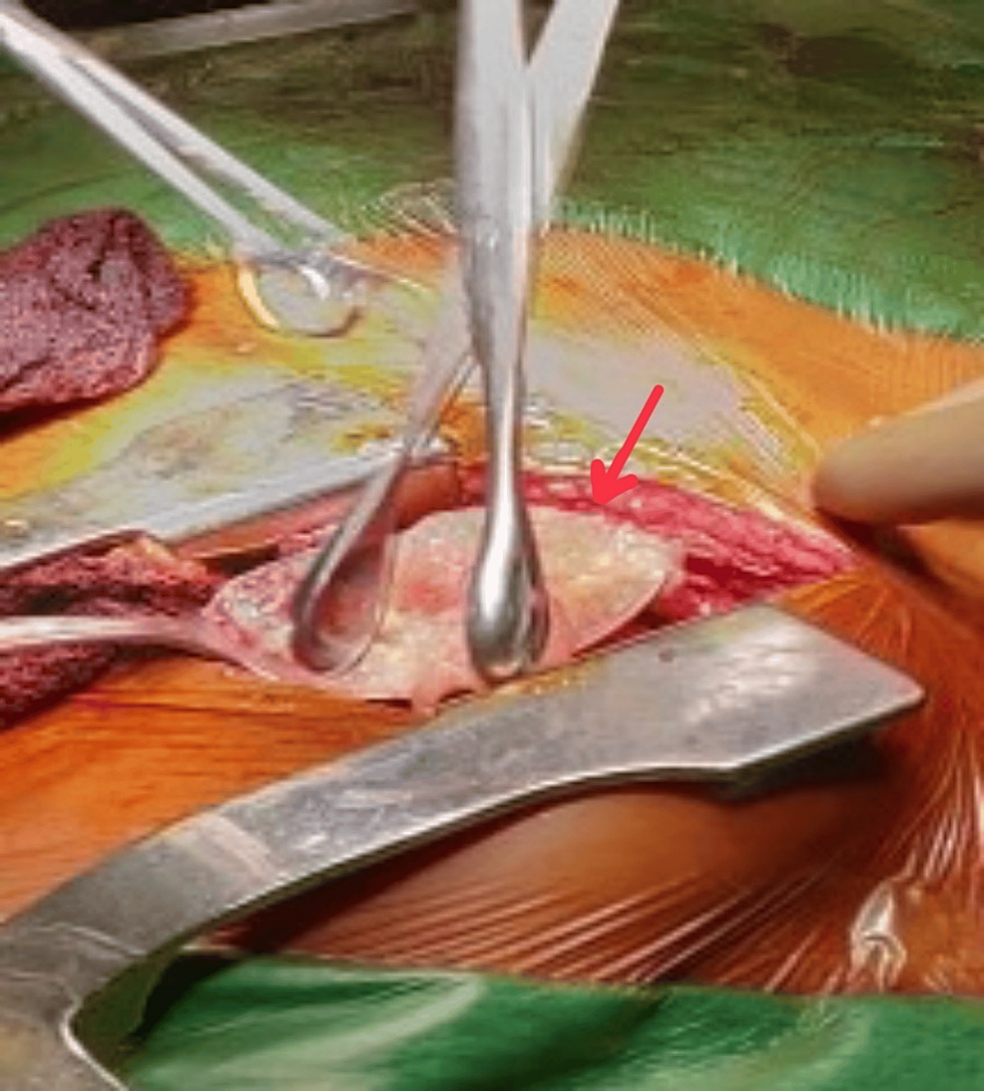
Intraoperative view of cyst extraction from the mediastinum.

After six weeks, the patient underwent a midline sternotomy and left anterolateral thoracotomy with excision of mediastinal cysts. Intraoperatively, there were two cysts in the anterior mediastinum, one measuring 10 cm × 8 cm which was completely excised, and the other was sized 5 cm × 8 cm, for which partial excision was done as it was found to be adherent with the lung and pericardium. Three cysts measuring 5 cm × 5 cm were also removed from the right costophrenic angle (Figure 5).

The patient was discharged on albendazole therapy and is on regular outpatient clinic follow-up. The patient had a prolonged intensive care unit stay postoperatively due to poor respiratory efforts. Macroscopically, the capsule of the cyst was intact, and the wall thickness of 0.2-0.3 cm on the cut section. The inner surface was gray-brown, glistening with focal gray-white areas with multiple daughter cysts varying in diameter from 0.5 to 1.5 cm with a wall thickness of 0.1 to 0.2 cm. Microscopically, ectocyst with inflammatory cell infiltrate was seen along with endocyst and brood capsule.

## Discussion

Hydatidosis or echinococcosis is a zoonotic larval infection that infects humans globally. *E. granulosus*, *E. multilocularis*, *E. vogeli*, and *E. oligarthus* are the species that are of concern in the human population. Intermediate hosts including humans demonstrate growth of hydatid cysts in the internal organs after larval infection of *E. granulosus*. Humans are affected by ingestion of food contaminated with the parasite eggs or by direct contact with an infected dog. The growth of cysts in humans is slow and over time forms a typical unilocular pattern of echinococcal cysts [2]. After entering the human intestines, the parasite enters the blood circulation and is responsible for its diverse locations. The most common site for hydatidosis is the liver (65%), followed by the lungs (25%), but can also affect other sites as well. Out of all, mediastinal localization is the rarest [3].

South America, Eastern Europe, Russia, the Middle East, and Central Asia are endemic to *E. granulosus*, where incidence rates are 50 per 100,000 person-years [4]. Hydatosis is responsible for the loss of 3.6 million disability-adjusted life years worldwide. In India, the disease is most prevalent in Andhra Pradesh and Tamil Nadu [5].

Infected hosts may stay symptom-free for months and years but its spontaneous rupture due to trauma or gradual increase in size can make the patient symptomatic. Multiple organ involvement is seen in 20-40% of cases. Signs and symptoms of the disease vary and depend on the size and site. Common findings in liver hydatidosis are abdominal pain, decreased appetite, hepatomegaly, a palpable mass, abdominal distension and chronic cough, chest pain, and shortness of breath in case of lung hydatidosis due to irritation of membranes [4].

A combination of appropriate history of exposure, examination, serological tests, and imaging helps in establishing the diagnosis of hydatidosis. In the case of liver hydatidosis, 40% of patients have deranged liver function tests showing elevated alkaline phosphatase and eosinophilia in complete blood count [6]. Elevated IgG levels may be seen in 60% of cases. The cysts might also cause suppression of the host’s immune system and the antibodies might be absent [7]. Circulating antibodies can be detected via immunoelectrophoresis, enzyme-linked immunosorbent assay (ELISA), and immunoblots. ELISA is used for screening purposes, while in case of a positive result, immunoblot assay for antigens is performed [8]. Imaging is helpful and the findings vary depending on the stage of the cyst and is important in seronegative cases. Fluid-filled cysts and calcifications can be seen on radiographs. Ultrasonography is the widely used, readily available, and cheap modality is the investigation of choice with 90-95% sensitivity. However, the location and number of cysts and the presence of daughter cysts require greater anatomic specificity which is achieved with the help of CT and MRI [9].

Treatment of hydatid disease can be medical or surgical. Surgical procedures can be either conservative or radical and the surgical approach depends on the case and can be posterolateral thoracotomy, anterolateral thoracotomy, or median sternotomy assisted with video-assisted thoracoscopic surgery in the case of lung hydatid and midline laparotomy in case of liver hydatid. The aim of the conservative procedure is sterilization, evacuation of cyst content, hydatidectomy and introduction of scolicidal agent, and then total aspiration. This approach is associated with complications such as anaphylactic reaction, secondary hydatidosis, and a high relapse rate of 20%. Radical procedures include removal of the cyst, pericystic membrane, and parasitic contents along with liver resection if indicated. They bear greater intraoperative risks but fewer complications with relapse rates. Puncture aspiration injection re-aspiration (PAIR) is an alternative to both surgery and medical management. It involves puncture of the cyst, aspiration of cyst fluid, injection of scolicidal agent, and re-aspiration of the cyst content. In case of relapse after PAIR and for cysts that are difficult to drain, percutaneous evacuation, modified catheterization technique, and dilatable multi-function trocar are preferred. Albendazole is the accepted medical therapy for hydatidosis and has better efficacy and absorption than mebendazole. However, its value in the definitive treatment of the disease remains controversial [10].

## Conclusions

Hydatid disease is a common disease in developing countries and usually involves one organ system. Mediastinal hydatid disease is a rare presentation and concurrent mediastinal with liver hydatid cysts is the rarest. Diagnosis is made by taking a thorough history combined with serology and imaging. Surgery is the definitive treatment.
